# A feasibility investigation of mindfulness-based cognitive therapy for people with Huntington’s disease

**DOI:** 10.1186/s40814-020-00631-z

**Published:** 2020-06-24

**Authors:** Fiona J. R. Eccles, David Craufurd, Alistair Smith, Rhys Davies, Kristian Glenny, Max Homberger, Siofra Peeren, Dawn Rogers, Leona Rose, Zara Skitt, Rachael Theed, Jane Simpson

**Affiliations:** 1 Division of Health Research, Faculty of Health and Medicine, Lancaster University, Lancaster, LA1 4YT UK; 2 Division of Evolution and Genomic Sciences, School of Biological Sciences, Faculty of Biology, Medicine and Health, Manchester Academic Health Science Centre, University of Manchester, Manchester, M13 9PL UK; 3 Manchester Centre for Genomic Medicine, Manchester Academic Health Science Centre, Manchester University NHS Foundation Trust, Oxford Road, Manchester, M13 9WL UK; 4 The Walton Centre NHS Foundation Trust, Lower Lane, Fazakerley, Liverpool, L9 7LJ UK

**Keywords:** Huntington’s disease, Mindfulness, Depression, Anxiety, Stress

## Abstract

**Background:**

Huntington’s disease (HD) is an inherited neurodegenerative condition which affects movement, coordination and cognitive functioning. Psychological difficulties are commonly experienced; however, psychological interventions have been little researched with this population. We investigated the feasibility of conducting a randomised controlled trial (RCT) of mindfulness-based cognitive therapy (MBCT) with people with the HD genetic mutation, either pre-manifest (before onset of movement symptoms) or at an early disease stage. Specifically, we evaluated the willingness of participants to be recruited into and complete the intervention; the acceptability of the study measures in relation to completion; the feasibility of offering the standard MBCT course to people with HD; the acceptability of the intervention and the estimated effect sizes.

**Methods:**

Participants were recruited from two UK HD centres and took part in an 8-week course of MBCT, with three reunions throughout the following year. Stress, depression, anxiety, and mindfulness were measured pre-, mid-, and post-course, at 3 months and at 1 year. Sleep, quality of life, positive affect and coping were measured pre- and post-course, at 3 months and at 1 year. Descriptive data and approximate effect sizes were calculated. Interviews were conducted post-course and at 1 year and data pertaining to the acceptability of the course were extracted.

**Results:**

Twelve participants took part in two groups; all were pre-manifest. Levels of depression and anxiety were low pre-course leaving little room for improvement. Changes in stress and in some aspects of mindfulness were medium to large. The qualitative data suggested participants rated the course highly and found it helpful and no changes to the standard course were needed. Recruitment levels were below those anticipated. Most measures were found to be acceptable.

**Conclusions:**

Although the course was acceptable to those who took part, given the difficulties in recruiting and the rarity of HD, conducting an RCT of MBCT teaching groups in person does not seem feasible. However, alternative modes of course delivery (e.g. online) would allow the recruitment of people from a greater geographical area and may make an RCT feasible; this revised focus would be suitable for future feasibility studies.

**Trial registration:**

ClinicalTrials.gov identifier NCT02464293, registered 8 June 2015.

## Background

Huntington’s disease (HD) is an inherited neurodegenerative condition, which affects movement, coordination and cognitive functioning, with emotional difficulties also commonly experienced. It is believed to affect as many as 12.3 per 100,000 people of European descent [1]. Each child of an affected person has a 50% chance of inheriting the condition; diagnosis, however, is not confirmed by a positive genetic test but on the onset of motor problems, which can be decades later. Age of diagnosis is typically around 35-55, with time from diagnosis to death around 20 years [2]. Many people at various stages of HD (including those who carry the gene mutation but are pre-manifest) experience low mood, anxiety and other psychological difficulties (e.g. [3–6]). Indeed, alongside functional capacity, depression and anxiety may be the main factors, which contribute to reduced health-related quality of life, more so than discrete motor problems, or cognitive impairment [7]. In addition, reports from patients suggest emotional and social concerns are important for individuals with the condition at the pre-manifest stage (e.g. anxiety about symptoms and impact of the condition on self and family, difficulties with acceptance of the condition and self-confidence, lack of support, perceived negative attitudes of others and limited public awareness) and similar concerns remain throughout the disease course [8]. Medication may be effective to alleviate psychological difficulties for some people [9], but its efficacy has not been conclusively proven [10] and it is not suitable for all. Furthermore, at least some would prefer not to take it, or to prolong the period without it [11].

Although it is commonly presumed that biological factors are the main determinants of psychological distress in people with HD, several studies have indicated that, while these may indeed be important, psychological factors are also significant. For example, both beliefs about the illness and coping mechanisms have been shown to be significant predictors of individuals’ mental health [12–14]. Such psychological beliefs and coping patterns can be adaptively changed using psychological interventions. For example cognitive-based psychological therapies such as cognitive behavioural therapy (CBT) presume cognitions and appraisals are key in experiences of physical illness and associated distress and that these, along with associated coping patterns, can be modified [15]. Such propositions are supported to some extent by empirical studies that have documented changes in beliefs, appraisals and coping strategies, some of which mediate outcomes in a variety of health conditions following CBT, e.g. [16–24].

However, little progress has been reported on the development of psychological interventions in HD despite the fact that people with HD have expressed an interest in psychological approaches [25] and these are currently being successfully developed for people with other neurological conditions with low mood, e.g. Parkinson’s disease (PD) and multiple sclerosis (MS) [26]. Indeed, currently, no feasibility study or pilot has been published in HD using any specific psychological therapy for psychological outcomes. Despite this, a recent qualitative study indicated that individuals with the HD gene who were receptive to the idea of psychological therapy did feel that non-pharmacological options should be open to them [11].

While a number of psychological approaches might be useful for individuals affected by HD, this study reports a feasibility assessment of mindfulness-based cognitive therapy (MBCT) for depression, with anxiety and stress, also considered as outcome measures. This particular therapy combines mindfulness practises with elements of cognitive therapy. During the course, individuals are taught to focus their attention in a particular way and to be present in the moment, bringing a non-judgemental stance to thoughts, feelings and experiences [27]. MBCT is most usually offered in a group format, with eight sessions of 2 h, with reunion meetings also available after the end of the course. The group format is commonly reported as providing useful support (e.g. [28]) and it is not unusual for groups to maintain contact after the formal course has ended.

Through engaging in MBCT, it is hypothesised that individuals become aware of unhelpful thought processes and beliefs, learn to decentre and disengage from them by focusing on present moment experiences, become aware that thoughts and feelings are temporary mental events rather than facts, and relate to their self and experiences more compassionately [29]. By standing back and bringing awareness and acceptance to experiences including distress, greater emotional regulation as well as emotional, behavioural and cognitive flexibility can be achieved and as a result, a greater (and more adaptive) range of coping strategies can be employed [30].

Although originally developed to help reduce relapse rates amongst people with prior recurrent depression [31, 32], MBCT has been increasingly used to help people with current difficulties [33–35]. Some, albeit limited, empirical evidence exists for the proposed mechanisms [29] including increased cognitive reappraisal ability (i.e. ability to change how one thinks about an emotional stimulus) [36], reduced reactivity to social stress [37] and a reduction in disengagement coping styles [38]. Qualitative findings suggest MBCT can lead to changed patterns of coping [39], with an increased sense of personal agency and control [28]. MBCT and a similar programme, mindfulness-based stress reduction (MBSR) [40], have been investigated in other neurological conditions where low mood and anxiety, for example, may also be the result of neurological changes as well as psychological and social factors, where individuals are adjusting to a physical health problem, and where difficulties with movement and cognition may also be present. For example, in a pilot RCT with people with PD, a programme based on MBCT reduced depression [41] and participants with PD have also reported improvements in self-management and psychological well-being following MBCT [39]. Programmes based on MBSR or MBCT for people with MS have shown improvements in a variety of outcomes including depression, anxiety and stress [42]. One study on MBSR for people with motor neuron disease/amyotrophic lateral sclerosis found improvements in a number of measures of distress [43]. Modifications have sometimes been necessary, for example, to reduce the physical component of the course (mindful movement) or reduce cognitive burden, e.g. [41, 44].

Feasibility studies are an increasingly useful way of indicating whether an intervention is acceptable and whether study parameters are practical. Given the lack of psychological interventions for individuals with HD, it was considered that a feasibility study was needed first to ascertain whether a future randomised controlled trial (RCT) of MBCT could be undertaken. The aim of this study was therefore to test the feasibility of conducting a future RCT on MBCT for people with the HD gene expansion who were pre-manifest or at an early disease stage.

## Methods

### Design

This study was designed to test the feasibility of a future RCT. Recent guidance [45] suggests that feasibility studies (as opposed to pilot studies) do not have to be randomised and should not just be a version of the intended randomised controlled trial with a smaller *N*, i.e. the study ‘in miniature’. In particular, this study set out to assess the willingness of participants to be recruited into and complete the intervention; the acceptability of the study measures in relation to completion; the feasibility of offering a group intervention to people with HD following the standard MBCT format; the acceptability of the intervention and the estimated effect sizes.

The study adopted a pre-post single group design with an embedded qualitative component, with follow-up up to a year. The main quantitative outcomes of interest were depression, anxiety and stress, along with mindfulness. Additional outcomes assessed were positive affect, sleep, coping and quality of life. However, as it is typical for feasibility studies [46], the study was not powered to detect significant changes, rather the data were used descriptively and to provide an estimate of effect sizes.

The qualitative data collected provided information on acceptability. An in-depth analysis of the participants’ experience will be reported in a separate publication.

### Participants

Participants had to be aged 18 or over and had to have been tested and found to have the relevant mutation on the huntingtin gene, meaning they would go on to develop symptoms of the disease in the future (if they had not already). Participants could be pre-manifest or symptomatic at stage 1 (i.e. score 11 or 12 or 13 on the total functional capacity scale [47] which is part of the unified Huntington’s disease rating scale [48]. Being at stage 1 means individuals are still able to function at home and at work and handle their financial affairs and domestic responsibilities. Participants had to have clinical signs of depression or low mood, identified in their medical notes or other information recorded at their last clinic visit (i.e. the clinician’s impression was that low mood or depression was present). Participants were excluded if they had active suicidal intent. There had to be no significant changes in medication in the 6 weeks prior to starting the MBCT intervention.

### Sample size

We aimed to recruit 15 participants for an MBCT course for pre-manifest individuals and 15 participants for an MBCT course for those at an early disease stage, from two HD centres in the North of England. MBCT classes for research often have approximately 12 participants per class [49] and therefore we were aiming for similar numbers, allowing for some attrition. It is advisable that mindfulness groups are not too small—both to ensure they remain classes rather than more a group therapy and for cost-effectiveness [49].

### Recruitment procedure

Potential participants were invited to participate by a member of their clinical team when attending routine clinic appointments. In addition, where patients had already consented to be contacted about research, they were contacted by telephone, email or letter to be told about the research. Potential participants could then contact the research team themselves to hear more about the research or give their consent for the clinical team to pass their details on to the research team who then contacted the potential participant. The mindfulness teacher described the nature of the course and what it entailed, and if they still wished to participate, a member of the research team met with them at home or another convenient location to take consent and complete the baseline measures.

### Intervention

The MBCT course was taught according to guidance in the manualised version of MBCT [49] by a registered mindfulness teacher. The course involves eight classes of 2 h each, at weekly intervals as well as about an hour a day of home practise. As is common in trials [49], the recommended day of silent practise between classes 6 and 7 was omitted due to limited funding. MBCT involves learning how to be mindful via formal meditations including sitting meditations (becoming aware of the breath), the body scan and mindful movement, and more informal practises, which help participants become mindful in everyday activities. In the year following the course, three reunion meetings at 3-monthly intervals were offered where participants revisited the principles and practise of mindfulness, discussed any difficulties and recommitted to their practise [49].

### Feasibility objectives

The objectives of the study were as follows:

1. To ascertain the willingness of participants to be recruited into and complete the intervention, thus to examine issues of recruitment, retention of participants and attendance at the mindfulness courses
2. To ascertain acceptability of the study measures by examining any difficulties with completion
3. To ascertain whether it was feasible to offer the standard MBCT course to individuals with HD
4. To ascertain the acceptability of the intervention
5. To estimate effect sizes

### Outcome measures

Depression and anxiety were measured using the 14-item Hospital Anxiety and Depression Scale (HADS; [50]) and stress with the 7-item stress subscale of the short form of the Depression, Anxiety and Stress Scale (DASS; [51]). Mindfulness was measured by the 39-item Five Facets of Mindfulness Questionnaire (FFMQ; [52]). This scale has five subscales—observing, describing, acting with awareness, non-judging and non-reacting—which measure the different aspects of mindfulness and is one of the most widely used measure of mindfulness which captures these different dimensions [53]. Coping was measured by the brief (28-item) COPE [54]; sleep quality assessed using the 19-item Pittsburgh Sleep Quality Index (PSQI; [55]); positive affect from the 10-item positive affect subscale from the Positive and Negative Affect Schedule, asking about the previous week (PANAS; [56]); and quality of life measured using the 26-item abbreviated scale of the World Health Organisation Quality of Life Instrument (WHOQOL-BREF; [57, 58]).

All items were based on self-report and were collected using the above well-validated measures. All measures were administered pre-, post-, 3 months post- and one-year post-course. Depression, anxiety, stress and mindfulness were also administered mid-course.

### Quantitative analysis

Quantitative data were tabulated for descriptive purposes. Given the small sample size, a non-parametric approach was used to calculate effect size data (*r* = *Z*/√ (*N*)), where *Z* is the standardised test-statistic from a Wilcoxon signed-rank test and *N* is the sample size [59], along with the associated 95% confidence interval. The *r* values are calculated for the timepoint as compared to baseline.

### Qualitative data

Qualitative interviews were completed with participants immediately post-course and one-year post-course to explore acceptability. An approach guided by framework analysis [60] was used to extract themes relevant to the issue of acceptability. This approach is useful for answering pragmatic evaluative questions [60].

## Results

### Recruitment rate

As pharmaceutical trials which particularly targeted those with early stage HD were ongoing at the same time as this study, the number of potential participants identified by referring clinicians was not as high as originally anticipated. By October 2015, we had recruited sufficient individuals to go ahead with one course—these were all pre-manifest individuals (cohort 1). Acceptance was approximately 1 in 3 of those approached. Three people who initially expressed interest could not attend (one not contactable, one could not do the day we had considered most accessible for most people—a Saturday—and one lived too far away). Eleven people started the course and one left after 1 session (due to a non-related health issue), the other 10 completed.

As only 10 participants had completed the first course and they were all pre-manifest, we decided to recruit for a second course starting October 2016, allowing the entry of both manifest and pre-manifest individuals. However, recruiting for a second group proved much more challenging. Most eligible pre-manifest patients who were interested had already taken part in the first cohort. We decided to involve a second smaller centre (the one geographically closest to the larger centre) to try to increase the potential pool. Unfortunately, this had fewer eligible potential participants than had been anticipated (eight in total).

Thus for cohort 2, two centres were involved. From the first centre, from the participants identified as eligible, five individuals expressed an interest and of these two were then not contactable. Two withdrew before the first day (one actively withdrew and one did not attend), one took part and completed. From the second centre, of the eight eligible individuals, four were interested. One person was symptomatic and felt the MBCT course location was too far to travel to and one person withdrew before the course started. One individual attended one session then withdrew (feeling it was not for them) and one person continued and completed.

As we were finding it hard to recruit sufficient numbers for cohort 2, participants from cohort 1 were also invited to return for a second course and three people accepted this invitation. It is not unusual for people to repeat mindfulness training [40] and this is often of further benefit to the person themselves as well as to others new to the approach. As one of the new participants from the second cohort commented: “it was quite good having some people doing it for the second time ‘cos you could tell they’d picked up a lot from the first time and they had like useful tips and things and that was good”. Three repeaters started the course and two completed (one withdrew due to a non-related health issue).

**Fig. 1:**
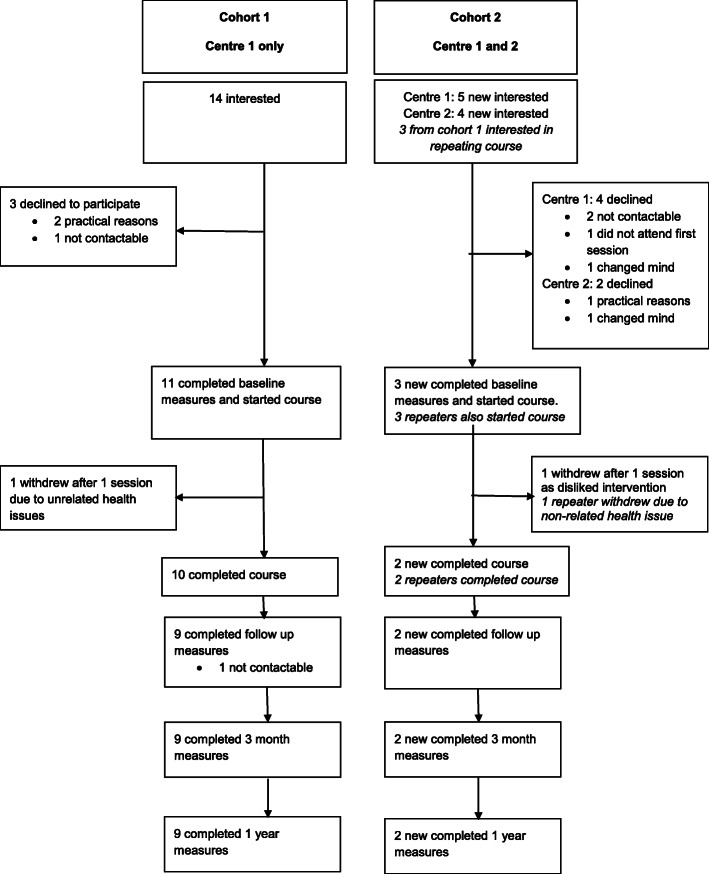
Flow of participants throughout the research. Repeaters in cohort 2 had completed the intervention in cohort 1 and took part in the intervention a second time. Only data for the new completers in cohort 2 (not the repeaters) are included in the results section

Thus, in summary, cohort 2 had six participants who started the course; three new participants and three participants from cohort 1 who opted to repeat the course and four participants completed (two new participants and two repeaters). The flow of participants can be seen in Fig. 1.

Generally, reasons given for non-participation included scheduling (the course was run on a weekend, to allow more people with work commitments to participate, however, this meant that some parents of young children could not participate due to childcare difficulties and others were working) and travel distance. Other patients suggested it was too difficult to attend 8 weeks of 2 h sessions because of other commitments. Some people were not interested, some could not conceive that the course would be of benefit and some did not wish to take part in a group intervention.

### Participants

The data presented are based on 12 participants who completed the course (10 from cohort 1 and the two new completers from cohort 2). Demographics of those who completed the course are given in Table 1.

**Table 1:** Demographics and clinical information

Age mean (SD), range	47 (11.7), 24-64
Gender (*n*)	
Male	4
Female	8
Ethnicity (*n*)	
White British	11
White Irish	1
Employment (*n*)	
Full time	6
Part time	2
Not employed or retired	4
Education (*n*)	
Degree or similar	3
A levels	2
School leavers’ cert.	1
O’ levels/GCSEs	4
No formal qualifications	2
Time since genetic test in years Mean (SD), range	9 (7.4), 1-21
On antidepressant medication	
Yes	7
No	5

Eight females and 4 males completed the MBCT course; all identified as White British or White Irish. They were aged 24-64 years (*M =* 47 years; *SD =* 12 years), with 6 in full-time employment, 2 in part-time employment and 4 not currently employed or retired. Regarding highest education, three had degree-level qualifications or similar, 2 A levels, 1 a school leavers certificate, 4 O’ levels/GCSE or similar and 2 no formal qualifications.

All participants were pre-manifest, in the sense that none had manifest motor signs of HD. According to their self-report, at the start of the course, the time since their genetic testing ranged from 1-21 years (*M =* 9 years) and seven participants were taking antidepressant medication.

As can be seen from Table 2, despite an inclusion criterion which stipulated that potential candidates should have expressed some difficulties with mood, most participants scored relatively low on the mood outcomes pre-intervention.

**Table 2:** Anxiety, depression, stress and mindfulness

	Pre		Mid				Post				3 months post				1 year post			
	*M*	SD	*M*	SD	*r*	*r* (CI)	*M*	SD	*r*	*r* (CI)	*M*	SD	*r*	*r* (CI)	*M*	SD	*r*	*r* (CI)
HADS-A	9.92	5.07	9.00	4.07	−0.25	−0.72, 0.38	8.18	4.31	−0.39	−0.80, 0.28	9.00	4.75	−0.14	−0.68, 0.50	8.27	3.17	−0.37	−0.79, 0.30
HADS-D	5.42	5.28	5.83	3.90	0.21	−0.42, 0.70	5.45	5.75	0.17	−0.48, 0.70	5.73	4.71	0.12	−0.51, 0.67	5.00	3.79	0.08	−0.55, 0.65
DASS-S	21.16	9.44	17.20	9.00	−0.41	−0.83, 0.29	14.90	9.44	−0.42	−0.81, 0.25	15.64	8.28	−0.54	−0.86, 0.09	13.8	8.24	−0.66	−0.91, −0.05
FFMQ																		
Observing	25.67	5.69	26.67	4.48	0.24	−0.39, 0.71	29.00	5.31	0.38	−0.28, 0.80	30.27	3.88	0.55	−0.07, 0.86	30.8	3.97	0.66	0.10, 0.90
Describing	23.92	6.43	26.83	6.29	0.43	−0.19, 0.80	27.73	6.74	0.80	0.39, 0.95	26.73	8.33	0.45	−0.21, 0.83	26.8	7.70	0.43	−0.23, 0.82
Acting with awareness	22.08	9.10	24.08	5.96	0.35	−0.28, 0.77	25.55	7.54	0.29	−0.37, 0.76	26.18	7.55	0.62	0.03, 0.89	27.4	7.65	0.63	0.05, 0.89
Non-judging	20.75	6.21	24.25	6.03	0.58	0.00, 0.86	25.64	6.27	0.60	0.01, 0.88	27.64	7.43	0.75	0.28, 0.93	28.0	6.15	0.85	0.50, 0.96
Non-reacting	16.58	5.33	21.33	3.60	0.69	0.20, 0.91	22.91	4.57	0.82	0.42, 0.95	22.64	3.70	0.77	0.31, 0.94	24.1	3.14	0.85	0.50, 0.96

Pre *n =* 12*;* mid *n =* 12 (except DASS-S, *n =* 10); post *n =* 11*;* 3-months *n =* 11*;* 1-year *n =* 11 (except DASS-S *n =* 10)

*HADS-A* hospital anxiety and depression scale-anxiety score; *HADS-D* hospital anxiety and depression scale-depression score; *DASS-S* depression anxiety and stress scale-stress score; *FFMQ* Five Factor Mindfulness Questionnaire. *r* is effect size (*r* = *Z*/√ (*N*), where *Z* is the standardised test-statistic from a Wilcoxon signed rank test and *N* is the sample size. *r* (CI) is 95% confidence interval for *r*. A negative *r* indicates improvement in depression, anxiety and stress and a positive *r* indicates improvement in mindfulness. DASS-21 stress scores are doubled to enable comparison with DASS-42

### Intervention and attendance

No adaptations to the standard course were made. When doing mindful movement, participants were advised not to undertake anything that could cause physical harm (e.g. not to walk too slowly if they were concerned about their balance).

Attending a minimum of five classes was considered evidence of course completion. Of the 14 participants who started classes, 12 met this criterion for completion (the other two left after one class).

### Measures completion

No difficulties were reported by the 12 participants with the measures of depression, anxiety, stress, mindfulness, coping, sleep and positive affect. For the HADS and FFMQ completion, numbers were as follows: pre *n =* 12; mid *n =* 12; post *n =* 11; 3-months *n =* 11; 1-year *n =* 11. For the DASS stress subscale, completion numbers were pre *n =* 12; mid *n =* 10; post *n =* 11; 3-months *n =* 11; 1-year *n =* 10. For the brief COPE completion numbers were pre *n =* 12; post *n =* 11; 3-months *n =* 11; 1-year *n* = 11, for the PSQI pre *n =* 12; post *n =* 11; 3-months *n =* 11; 1-year *n* = 10 and for the PANAS positive subscale pre *n =* 12; post *n =* 10; 3-months *n =* 11; 1-year *n* = 11.

However, only nine participants completed the quality of life measure at baseline. The use of this measure requires that data are shared with the measure’s authors and includes the participant’s date of birth and this was made clear in the consent process. As a result, three participants opted not to complete it. Completion numbers were pre *n =* 9; post *n =* 8; 3-months *n =* 8; 1-year *n = 8.*

### Effect sizes

A descriptive summary of the outcomes can be seen in Table 2 and Table 3 along with effect sizes.

**Table 3:** Positive affect, coping, sleep and quality of life

	Pre		Post				3 months post				1 year post			
	*M*	SD	*M*	SD	*r*	*r* (CI)	*M*	SD	*r*	*r* (CI)	*M*	SD	*r*	*r* (CI)
PANAS-P	29.2	9.0	31.9	10.2	−0.11	−0.69, 0.56	30.5	6.9	0.16	−0.49, 0.69	32.2	8.1	0.35	−0.32, 0.78
COPE														
Self-distraction	5.50	1.89	4.91	2.17	−0.28	−0.75, 0.38	4.55	2.02	−0.39	-0.80, 0.28	4.64	2.16	−0.38	−0.80, 0.28
Active coping	5.25	1.66	5.73	2.00	0.42	−0.24, 0.81	5.64	1.86	0.13	−0.51, 0.68	6.00	1.90	0.30	−0.37, 0.76
Denial	3.58	1.93	2.73	1.19	−0.52	−0.85, 0.12	2.64	1.50	−0.51	−0.85, 0.12	2.45	0.94	−0.60	-−0.88, 0.00
Substance use	3.17	2.12	3.27	2.05	0.00	−0.60, 0.60	3.64	1.91	0.39	−0.27, 0.80	3.45	2.16	0.30	−0.36, 0.76
Emotional support	4.67	2.39	5.27	1.85	0.39	−0.28, 0.80	5.00	2.28	0.11	−0.53, 0.66	5.55	1.81	0.34	−0.33, 0.78
Instrumental support	4.58	2.23	4.82	1.89	0.26	−0.40, 0.74	3.91	1.81	−0.56	−0.87, 0.06	5.09	1.64	0.16	−0.48, 0.70
Behavioural disengagement	4.00	1.60	3.18	1.89	−0.53	−0.86, 0.10	2.55	1.04	−0.64	−0.90, −0.07	2.82	1.17	−0.60	−0.88, 0.00
Venting	4.50	1.93	3.90	1.45	−0.55	−0.86, 0.08	4.00	1.34	−0.16	−0.70, 0.48	3.91	1.70	−0.26	−0.74, 0.40
Positive reframe	4.83	1.53	5.55	1.92	0.44	−0.21, 0.82	5.36	1.69	0.28	−0.39, 0.75	6.27*	1.62	0.61	0.01, 0.89
Planning	4.83	1.90	5.45	2.34	0.47	−0.18, 0.83	4.73	1.90	−0.08	−0.65, 0.55	4.91	2.02	0.00	−0.60, 0.60
Humour	4.92	2.19	5.00	2.37	0.16	−0.49, 0.69	4.55	2.02	−0.26	−0.74, 0.40	4.73	2.24	−0.20	−0.71, 0.46
Acceptance	6.75	1.42	6.27	2.01	−0.24	−0.73, 0.42	5.82	1.47	−0.57	−0.87, 0.05	6.64	2.16	−0.06	−0.63, 0.56
Religion	3.67	2.42	4.91	2.26	0.74	0.26, 0.93	4.36	2.11	0.46	−0.19, 0.83	4.73	2.69	0.67	0.12, 0.91
Self-blame	4.42	1.56	3.91	1.51	−0.28	−0.75, 0.39	3.82	1.83	−0.26	−0.74, 0.41	3.18	1.32	−0.47	−0.83, 0.18
PSQI Total score	8.42	4.66	7.82	4.47	−0.28	−0.75, 0.39	6.36	3.70	−0.34	−0.78, 0.33	6.50	3.78	−0.36	−0.81, 0.35
WHOQOL														
Physical	61.1	25.1	64.3	28.1	−0.07	−0.70, 0.62	65.6	22.5	0.00	−0.70, 0.70	64.3	28.3	−0.18	−0.79, 0.60
Psychological	52.7	26.8	60.7	24.3	0.28	−0.47, 0.80	52.6	25.0	−0.24	−0.81, 0.56	56.5	17.6	0.00	−0.70, 0.70
Social	61.1	26.4	67.7	36.0	0.23	−0.52, 0.77	58.3	32.1	−0.40	−0.86, 0.42	62.5	31.2	0.00	−0.70, 0.70
Environmental	70.1	17.9	69.1	21.5	−0.03	−0.68, 0.65	67.2	23.8	−0.42	−0.87, 0.41	66.0	19.8	−0.48	−0.89, 0.34

Pre *n =* 12 (except WHOQOL *n =* 9)*;* post *n =* 11 (except PANAS-P *n =* 10; WHOQOL *n = 8*)*;* 3-months *n =* 11 (except WHOQOL *n = 8*); 1-year *n = 11* (except PSQI *n = 10; WHOQOL n = 8*)

*PANAS-P* positive subscale of the positive and negative affect scale. *PSQI* Pittsburgh Sleep Quality Index *WHOQOL* brief version of the World Health Organisation Quality of Life Instrument. *r* is effect size (*r* = *Z*/√ (*N*), where *Z* is the standardised test statistic from a Wilcoxon signed-rank test and *N* is the sample size. *r* (CI) is 95% confidence interval for *r*. A positive *r* indicates an increase in positive affect on the PANAS-P, an increase in quality of life on the WHOQOL and an increase in use of each coping strategy on the COPE. A negative *r* indicates improvement in sleep on the PSQI

Inspection of the effect sizes (0.1, small; 0.3, medium; 0.5, large) indicates that of the psychological distress outcomes (depression, anxiety and stress), the consistently larger effects were for stress. In relation to mindfulness, all effect sizes were in the predicted direction and for several of the subscales—e.g. non-judging and non-reacting—these were consistently large across time.

Changes were generally minimal for coping, sleep and positive affect. The main effect sizes of note are mainly in the COPE at 1 year, where they are large for decreases in behavioural disengagement and denial and increases in positive reframing and religious coping. However, the confidence intervals for all outcomes are wide so there is considerable uncertainty in these findings.

### Acceptability of the intervention

Eleven people took part in the immediate follow-up interviews and ten in those at 1 year. The following themes related to acceptability were extracted.

#### Overall appraisal of the classes

Everyone gave a positive appraisal of their experience of learning mindfulness and most enjoyed the classes.

The classes […] they were really good. They were helpful (P9, Immediate Follow Up, (FU))

I was quite surprised actually how enjoyable it was really and how helpful it was (P5, Immediate FU)

Each lesson I was learning something new about yourself a bit and about how your mind works (P4, Immediate FU)

#### Meeting others with HD

At recruitment, several people had expressed anxiety about meeting other participants who were symptomatic and thus seeing how the disease might develop. However, in the event, everyone who took part was pre-manifest (i.e. not yet showing motor symptoms), which was described as a relief. As HD is rare, most people appreciated the chance to meet with others who had the genetic mutation.

[The course] seemed really useful and it was good to meet other people in a similar situation (P22, immediate FU)

I mean, even just sitting in a group working together with people that have got HD as well as themselves, I think that alone is quite therapeutic (P8, immediate FU)

#### Reunions

The three reunions that were offered in the year following the course were also described by participants as beneficial, both for the group support and for providing an opportunity for people to renew their commitment to practising mindfulness, particularly if practise had fallen away over the preceding 3 months.

“I felt the support from the group, and the empathy from the group was very, very powerful.” (P1, one year FU)

I think they [the reunions] really did help to, yeah, get back on track and reconnect. (P22, 1-year FU)

#### Continuing practise

Following the end of the 8 weeks of classes, most people intended to continue to practise mindfulness, to some extent, whether that was informally in their everyday lives or more formally doing the specific mindfulness practises.

I’m feeling really strongly about mindfulness and I’m practising it mostly daily because I really feel it’s helped me (P10, Immediate FU)

I will definitely keep on with it. Whether or not I do it as frequently. I don’t know (P8, Immediate FU)

At 1 year, people were still using the skills, although some to a greater extent than others:

I’ve never gone more than a few days without doing it [formal mindfulness practice] (P1, 1-year FU)

It’s not like a daily thing. Just whenever I get stressed it just feels like it’s a quick way to get rid of any stress (P9, 1-year FU)

#### Practicalities

There was a general consensus that it was beneficial to meet off the hospital site. However, due to financial constraints, the venue was quite basic, with some issues with the heating (needed in the UK winter when participants are stationary) and some participants commented that an alternative, more pleasant venue would have been preferable.

#### Participants who took the course twice

Two participants who took and completed the course twice reported a particular benefit from the intervention. Not only did they attend the course twice, they also continued to attend both sets of reunion classes and both started attending mindfulness events outside of the research study. Both described learning mindfulness through the course as ‘life-changing’, with one person additionally describing it ‘one of the most profound experiences’ (P3, 1-year FU).

## Discussion

This study set out to investigate the feasibility of performing an RCT of MBCT with individuals with the HD gene who were pre-manifest or at an early disease stage. In particular, the aims were to assess the willingness of participants to be recruited into and complete the intervention; to assess the acceptability of the study measures; to assess the feasibility of offering a group 8 week intervention to people with HD; to assess the acceptability of the intervention and to estimate effect sizes. The findings will be discussed in relation to these specific aims as well as the broader issues which arose relating to a possible future RCT or future feasibility and pilot work.

### Challenges in recruitment

We were able to run two courses of MBCT. Cohort 1 consisted of 11 participants (10 completers) who were recruited from one large UK centre. Recruiting for this course was relatively successful, with uptake approximately 1 in 3 (i.e. approximately a third of eligible pre-manifest patients from the centre who were approached opted to take part). However, recruiting for the second course was much more difficult. Despite also involving a second smaller centre only three new participants started the second course (along with three repeating participants).

As is often the case, relationships were key, and recruitment was most successful when done in person via staff who knew the patients (or at least the patients and lead consultant had a good relationship and therefore patients were interested in research). Furthermore, in the large centre, the main recruiting staff had knowledge of mindfulness and were keen to promote it. Finally, perhaps recruitment was more straightforward in the larger centre where research participation was routine and embedded into clinic visits.

Recruiting a group of people with a relatively rare condition is challenging from a geographic perspective. Patients can travel over 2 hours to get to appointments at a main centre and to travel this distance on a weekly basis is a considerable burden. This is perhaps even more so when the early symptoms could include problems with fatigue or motivation and some mild cognitive problems. Finally, it can be hard for some people with a genetic medical condition to conceive that a psychological intervention could help their mood difficulties, particularly if they view them as an inevitable part of their condition [11], and thus considerable work is needed promoting the research and potential for psychological intervention.

The inclusion of individuals in cohort 2 who had completed cohort 1 was a pragmatic decision to ensure the class could run and to allow us to collect further information on feasibility. As noted above, repeating MBCT classes is not unusual and often happens in clinical practise. However, to our knowledge, the effect of repeaters on class outcomes has not been studied and therefore it is not possible to ascertain for sure if it affects outcomes. If conducting a full-sized RCT inclusion of repeaters would need to be considered and it may be prudent to include solely new participants.

This study recruited mainly from one of the largest HD centres in the UK. Considering future studies, a recruitment rate of 1 in 3 at a large HD centre would probably be sufficient to permit one course of MBCT to take place (with approximately 10-12 participants) with pre-manifest individuals. However, given the low rate of distress reported, at least on the measures adopted here (discussed further below), recruiting sufficient pre-manifest individuals reporting high levels of distress would not be feasible from one centre. Furthermore, if twice this number were required to permit a control group for an RCT, then recruiting sufficient numbers of pre-manifest individuals, regardless of distress, would not be possible from one centre. Recruiting individuals at an early disease stage was completely unsuccessful so currently, it appears unfeasible to do an RCT on MBCT recruiting this way for them.

### Acceptability of measures

Participants were able to complete most of the measures and no participants reported them as particularly burdensome. However, it would have been beneficial to capture other aspects of well-being, for example, apathy and irritability which have been discussed in relation to HD [3, 5]. Furthermore, the qualitative findings, along with discussions in the classes, suggested the benefits of participants experienced were not captured well in the quantitative measures. Future studies may benefit from considering outcomes other than anxiety and depression, and also considering capturing a broader understanding of well-being. Indeed focusing on positive outcomes, such as improving well-being or resilience, may be a more useful outcome for this group for future studies. Furthermore, instead of the COPE, which looks at a broad range of specific coping strategies, it may be preferable to target the coping strategies theorised as directly influenced by mindfulness. Some of these are captured in the FFMQ but other concepts that could be examined include problem-focused and approach-avoidance coping and experiential avoidance [38, 61] or coping flexibility [62].

A further issue relates to the use of the HADS. At the time of conception of the project, the HADS was recommended for identifying depression in people with HD [63] and was in use for other international large scale studies in HD, e.g. REGISTRY (http://www.euro-hd.net/html/registry) and ENROLL-HD (https://www.enroll-hd.org/). However, more recently, the factor structure has been questioned [64], including in HD [65], with suggestions that the total score may be more appropriate as a measure of distress [64] and that in HD only certain items should be included [65]. Thus, the use of these other scoring approaches should be considered for general distress and other measures of anxiety and depression may be better for measuring these outcomes specifically.

The one measure that caused some difficulty was the WHOQOL-BREF [57, 58]. Some participants opted not to complete it as they were uncomfortable with their data being shared with the measure’s authors and thus with individuals outside of the immediate research team. Furthermore, on reflection, the broad nature of this measure (including, for example, measuring the environmental quality of life) could reduce the sensitivity to change from this particular intervention. For future trials of this nature, an HD specific health-related quality of life measure, such as the HD Health-Related Quality of Life Questionnaire [66] may be more appropriate.

### Feasibility of a group 8-week MBCT intervention

The standard MBCT programme (without the all-day class) was able to be implemented without any changes. All participants who completed the course were able to attend the minimum of five sessions required for course completion. This would suggest that for pre-manifest individuals, the standard MBCT course could be appropriate for future work. However, given the low levels of distress (at least reported on the measures), it may be that a mindfulness-based intervention with a greater focus on increasing flourishing, rather than reducing depression, may warrant further exploration. Two such interventions also delivered as 8-week courses are MBSR and MBCT for life: the former has been extensively researched, while the latter, developed by prominent MBCT researchers and others, though promising is rather new, therefore less well established.

The exclusion of the all-day class was partly a pragmatic decision but is also common in research studies [49] although future pilot studies may wish to consider including this if the design permits (e.g. the contact time does not need to be controlled compared to another trial arm which can be problematic with this element). As the time commitment was already an issue for some participants here, the views of participants regarding this element may need to be sought in feasibility work.

Regarding the 8-week commitment, this was not problematic for those who attended; indeed, several commented that they would have preferred a longer timeframe. However, during recruitment, some potential participants declined due to difficulties relating to access and the time commitment required. Alternative ways of accessing the course which do not require attendance in person may need to be considered to make MBCT more accessible, such as a delivery via group online video call [67, 68] or a hybrid model with part delivered online and part in person. Such a delivery mode would also remove the need for all participants to live in the same geographical area and online recruitment methods could also be considered. Both of these would need exploring first in further feasibility work, particularly given the apparent importance of relationships and connection to the clinic mentioned during recruitment.

For those who took part in the intervention, the group format was a facilitator of completion and engagement. However, feedback from the study recruiters indicated that the group aspect did not appeal to some individuals and this was one of several factors that reduced enrolment. Group interventions are widely acknowledged to have the potential for dichotomising potential clients [69] with many participants gaining substantially from the group element and others finding the group setting overwhelming or uncomfortable, and this study provides further evidence of this. MBCT was originally designed as a group intervention and groups can also be cost-effective but individual mindfulness interventions are being explored in other health conditions (e.g. [70]), so this could be considered for future work. Generally, this highlights the need for a variety of therapies to be available for people with HD.

### Acceptability of the intervention

The participants who participated in the intervention were clearly willing to try a psychological intervention, despite the dominant narrative that psychological difficulties in HD are largely neurologically mediated. The effect sizes and qualitative data indicated that participants were able to learn mindfulness skills and the good attendance at classes and reunions and continued practise of mindfulness by participants suggest the intervention was acceptable to most of them. This suggests that pursuing mindfulness-based approaches for future work is worthwhile.

### Effect sizes

The calculation of effect sizes was complicated by a number of factors relating to recruitment. The levels of depression at the start of the course were actually relatively low as a group and thus there was little room for improvement (and therefore for effect sizes to be high). All participants had expressed some level of psychological distress in their last visit to the clinic, however, for some people their mood had improved since that visit (perhaps due to medication or a change in life situation) and for others, the measures did not appear consistently to reflect the participant’s state of mind. Indeed for one or two participants, their scores worsened over time as they became more aware of their mood (and verbally reported being more ‘in touch’ with how they were feeling). The difficulty of capturing increased insight in therapy (and thus sometimes worsening mood scores) is a common problem in therapeutic outcome (e.g. [71]). Finally, it is acknowledged that caution is needed when drawing any conclusions on effect sizes from feasibility studies [72]. Given these difficulties, alongside the other issues in this feasibility study, the current effect size calculations are probably not useful for powering future studies.

## Conclusions

In summary, it would not be feasible to perform an RCT following the precise method of the current study. However, a number of recommendations can be made, some of which could be further tested in future feasibility or pilot work.

- No symptomatic individuals were recruited and thus it may be that recruiting them for this type of intervention is not feasible. However, alternative avenues of recruitment for symptomatic individuals may be useful to investigate before this can be concluded.
- Group settings can be positive for many participants, but will not appeal to all and therefore individual psychological therapy might need to be available.
- Given the very positive experiences for those who were willing and able to attend, we think mindfulness-based therapy as an approach is an area of research that should be pursued further for individuals with the HD gene. We are particularly interested in trying to develop an approach that not only allows greater flexibility in terms of access but also retains some of the benefits of participants meeting face-to-face. It may be that a hybrid model with some face-to-face meetings but also taking elements of the current online courses available and/or apps may be a useful avenue to pursue.
- Alternatively, a supported online version of the MBCT course would allow a greater number of people access and so could cover a much wider geographical area and allow people to ‘attend’ in a more flexible way, and may be useful given the rarity of the condition. As part of this approach, it may be possible to recruit through online channels (e.g. via HD charities) which could broaden the potential participant pool. Such an approach would need feasibility work to explore the willingness of participants to be recruited outside of HD clinics and to explore whether participants found this mode of accessing therapy acceptable.
- While 8 weeks represents a considerable commitment and was not possible for some potential participants, we would be hesitant to suggest a shorter course as there is a risk that individuals might not be able to engage fully with the concepts in a shorter time. Indeed, many of the current participants reported that they thought the course could be longer.
- Individuals with the HD gene might also be able to access generic MBCT courses (and other similar mindfulness-based interventions) if available in their local area, whether through the NHS (in the UK) or registered private providers. However, given the very specific psychological challenges faced by this group—and also supported by the qualitative feedback we received from participants—much value resides in offering evidence-based interventions tailored to this group. As a compromise, it may be possible to run groups with individuals with conditions that are similar in terms of difficulties in movement, cognition, behaviour and psychological distress (e.g. Parkinson’s disease or Parkinson’s plus disorders), although this would need further research to investigate acceptability to participants and effectiveness.

## Data Availability

The datasets generated and/or analysed during the current study are not publicly available but are available from the corresponding author on reasonable request.

## Abbreviations

CBT: Cognitive behavioural therapy
DASS: Depression, Anxiety and Stress Scale
FFMQ: Five Factor Mindfulness Questionnaire
FU: Follow-up
HADS: Hospital Anxiety and Depression Scale
HD: Huntington’s disease
MBCT: Mindfulness-based cognitive therapy
MBSR: Mindfulness-based stress reduction
MS: Multiple sclerosis
NHS: National Health Service
PANAS: Positive and Negative Affect Schedule
PD: Parkinson’s disease
PSQI: Pittsburgh Sleep Quality Index
RCT: Randomised controlled trial
WHOQOL-BREF: Brief version of the World Health Organisation Quality of Life Instrument

## References

[1] Evans SJ, Douglas I, Rawlins MD, Wexler NS, Tabrizi SJ, Smeeth L (2013). Prevalence of adult Huntington’s disease in the UK based on diagnoses recorded in general practice records. J Neurol Neurosurg Psychiatry.

[2] Kremer B, Bates G, Harper P, Jones L (2002). Clinical neurology in Huntington’s disease. Huntington's disease.

[3] Craufurd D, Thompson JC, Snowden JS (2001). Behavioral changes in Huntington disease. Cogn Behav Neurol.

[4] Julien CL, Thompson JC, Wild S, Yardumian P, Snowden JS, Turner G (2007). Psychiatric disorders in preclinical Huntington’s disease. J Neurol Neurosurg Psychiatry.

[5] Paulsen JS, Ready RE, Hamilton JM, Mega MS, Cummings JL (2001). Neuropsychiatric aspects of Huntington’s disease. J Neurol Neurosurg Psychiatry.

[6] Craufurd D, Snowden JS, Bates G, Tabrizi SJ, Jones L (2014). Neuropsychiatry and neuropsychology. Huntington’s disease.

[7] Ho AK, Gilbert AS, Mason SL, Goodman AO, Barker RA (2009). Health-related quality of life in Huntington’s disease: which factors matter most?. Mov Disord.

[8] Ho AK, Hocaoglu MB (2011). For the European Huntington’s disease network quality of life working G. impact of Huntington’s across the entire disease spectrum: the phases and stages of disease from the patient perspective. Clin Genet.

[9] Videnovic A (2013). Treatment of Huntington disease. Curr Treat Options Neurol.

[10] Mestre TA, Ferreira JJ (2012). An evidence-based approach in the treatment of Huntington’s disease. Parkinsonism Relat D.

[11] Theed R, Eccles FJR, Simpson J (2018). Understandings of psychological difficulties in people with the Huntington’s disease gene and their expectations of psychological therapy. Psychol Psychother-Theory Res Pract..

[12] Helder DI, Kaptein AA, Kempen GMJ, Weinman J, Houwelingen HC, Roos RAC (2002). Living with Huntington’s disease: illness perceptions, coping mechanisms, and patients’ well-being. Brit J Health Psych.

[13] Kaptein AA, Scharloo M, Helder DI, Snoei L, van Kempen GMJ, Weinman J (2007). Quality of life in couples living with Huntington’s disease: the role of patients’ and partners’ illness perceptions. Qual Life Res.

[14] Arran N, Craufurd D, Simpson J (2013). Illness perceptions, coping styles and psychological distress in adults with Huntington’s disease. Psychol Health Med.

[15] Eagle A, Worrell M, Llewellyn CD, McManus C, Weinman J, Petrie KJ, Newman S, Ayers S (2019). Cognitive behaviour therapy. Cambridge handbook of psychology, health and medicine. Cambridge handbooks in psychology.

[16] Chilcot J, Moss-Morris R (2013). Changes in illness-related cognitions rather than distress mediate improvements in irritable bowel syndrome (IBS) symptoms and disability following a brief cognitive behavioural therapy intervention. Behav Res Ther.

[17] Christensen SS, Frostholm L, Ørnbøl E, Schröder A (2015). Changes in illness perceptions mediated the effect of cognitive behavioural therapy in severe functional somatic syndromes. J Psychosom Res.

[18] Goodman D, Morrissey S, Graham D, Bossingham D (2005). The application of cognitive-behaviour therapy in altering illness representations of systemic lupus erythematosus. Behav Chang.

[19] Renn BN, Hundt NE, Sansgiry S, Petersen NJ, Kauth MR, Kunik ME (2018). Integrated brief cognitive behavioral therapy improves illness intrusiveness in veterans with chronic obstructive pulmonary disease. Ann Behav Med.

[20] Knoop H, van Kessel K, Moss-Morris R (2012). Which cognitions and behaviours mediate the positive effect of cognitive behavioural therapy on fatigue in patients with multiple sclerosis?. Psychol Med.

[21] Dobkin RD, Menza M, Allen LA, Gara MA, Mark MH, Tiu J (2011). Cognitive-behavioral therapy for depression in Parkinson’s disease: a randomized, controlled trial. Am J Psychiatry.

[22] Kiropoulos LA, Kilpatrick T, Holmes A, Threader J (2016). A pilot randomized controlled trial of a tailored cognitive behavioural therapy based intervention for depressive symptoms in those newly diagnosed with multiple sclerosis. BMC Psychiatry.

[23] Morley S, Eccleston C, Williams A (1999). Systematic review and meta-analysis of randomized controlled trials of cognitive behaviour therapy and behaviour therapy for chronic pain in adults, excluding headache. Pain..

[24] Goldstein LH, McAlpine M, Deale A, Toone BK, Mellers JDC (2003). Cognitive behaviour therapy with adults with intractable epilepsy and psychiatric co-morbidity: preliminary observations on changes in psychological state and seizure frequency. Behav Res Ther.

[25] Aoun S, Kristjanson L, Oldham L (2006). The challenges and unmet needs of people with neurodegenerative conditions and their carers. J Commun Nurses.

[26] Ghielen I, Rutten S, Boeschoten RE, Houniet-de Gier M, van Wegen EEH, van den Heuvel OA (2019). The effects of cognitive behavioral and mindfulness-based therapies on psychological distress in patients with multiple sclerosis, Parkinson’s disease and Huntington’s disease: two meta-analyses. J Psychosom Res.

[27] Williams JMG, Teasdale JD, Segal ZV, Kabat-Zinn J (2007). The mindful way through depression.

[28] Cairns V, Murray C (2013). How do the features of mindfulness-based cognitive therapy contribute to positive therapeutic change? A meta-synthesis of qualitative studies. Behav Cogn Psychother.

[29] van der Velden AM, Kuyken W, Wattar U, Crane C, Pallesen KJ, Dahlgaard J (2015). A systematic review of mechanisms of change in mindfulness-based cognitive therapy in the treatment of recurrent major depressive disorder. Clin Psychol Rev.

[30] Shapiro SL, Carlson LE, Astin JA, Freedman B (2006). Mechanisms of mindfulness. J Clin Psychol.

[31] Ma SH, Teasdale JD (2004). Mindfulness-based cognitive therapy for depression: replication and exploration of differential relapse prevention effects. J Consult Clin Psychol.

[32] Teasdale JD, Segal ZV, Williams JMG, Ridgeway VA, Soulsby JM, Lau MA (2000). Prevention of relapse/recurrence in major depression by mindfulness-based cognitive therapy. J Consult Clin Psychol.

[33] Eisendrath SJ, Delucchi K, Bitner R, Fenimore P, Smit M, McLane M (2008). Mindfulness-based cognitive therapy for treatment-resistant depression: a pilot study. Psychother Psychosom.

[34] Barnhofer T, Crane C, Hargus E, Amarasinghe M, Winder R, Williams JMG (2009). Mindfulness-based cognitive therapy as a treatment for chronic depression: a preliminary study. Behav Res Ther.

[35] Teasdale JD, Williams JMG, Segal ZV (2014). The mindful way workbook: an 8-week program to free yourself from depression and emotional distress.

[36] Troy AS, Shallcross AJ, Davis TS, Mauss IB (2013). History of mindfulness-based cognitive therapy is associated with increased cognitive reappraisal ability. Mindfulness..

[37] Britton WB, Shahar B, Szepsenwol O, Jacobs WJ (2012). Mindfulness-based cognitive therapy improves emotional reactivity to social stress: results from a randomized controlled trial. Behav Ther.

[38] Cousin G, Crane C (2016). Changes in disengagement coping mediate changes in affect following mindfulness-based cognitive therapy in a non-clinical sample. Br J Psychol.

[39] Fitzpatrick L, Simpson J, Smith A (2010). A qualitative analysis of mindfulness-based cognitive therapy (MBCT) in Parkinson’s disease. Psychol Psychother-Theory Res Pract.

[40] Kabat-Zinn J (2013). Full catastrophe living. How to cope with stress, pain and illness using mindfulness meditation. 2nd ed.

[41] Rodgers SH, Schütze R, Gasson N, Anderson RA, Kane RT, Starkstein S (2019). Modified mindfulness-based cognitive therapy for depressive symptoms in Parkinson’s disease: a pilot trial. Behav Cogn Psychother.

[42] Simpson R, Simpson S, Ramparsad N, Lawrence M, Booth J, Mercer SW (2019). Mindfulness-based interventions for mental well-being among people with multiple sclerosis: a systematic review and meta-analysis of randomised controlled trials. J Neurol Neurosurg Psychiatry.

[43] Pagnini F, Marconi A, Tagliaferri A, Manzoni GM, Gatto R, Fabiani V (2017). Meditation training for people with amyotrophic lateral sclerosis: a randomized clinical trial. Eur J Neurol.

[44] Simpson R, Byrne S, Wood K, Mair FS, Mercer SW (2018). Optimising mindfulness-based stress reduction for people with multiple sclerosis. Chronic Illness.

[45] Arain M, Campbell MJ, Cooper CL, Lancaster GA. What is a pilot or feasibility study? A review of current practice and editorial policy.(Correspondence)(Editorial). BMC Med Res Methodol. 2010;10:67. 10.1186/1471-2288-10-67 PMC2912920 20637084

[46] Billingham SA, Whitehead AL, Julious SA (2013). An audit of sample sizes for pilot and feasibility trials being undertaken in the United Kingdom registered in the United Kingdom clinical research network database. BMC Med Res Methodol.

[47] Shoulson I, Kurlan R, Rubin AJ, Munsat TL (1989). Assessment of functional capacity in neurodegenerative movement disorders: Huntington’s disease as a prototype. Quantification of Neurological Deficit.

[48] The Huntington’s Study Group (1996). Unified Huntington’s disease rating scale: reliability and consistency. Mov Disord.

[49] Segal ZV, Williams JMG, Teasdale JD. Mindfulness-based cognitive therapy for depression. 2nd ed. New York: New York: Guilford Press; 2013.

[50] Zigmond AS, Snaith RP (1983). The Hospital Anxiety and Depression Scale. Acta Psychiatr Scand.

[51] Lovibond SH, Lovibond PF (1995). Manual for the depression, anxiety, stress scales.

[52] Baer RA, Smith GT, Hopkins J, Krietemeyer J, Toney L (2006). Using self-report assessment methods to explore facets of mindfulness. Assessment..

[53] Sauer S, Walach H, Schmidt S, Hinterberger T, Lynch S, Büssing A (2013). Assessment of mindfulness: review on state of the art. Mindfulness..

[54] Carver CS (1997). You want to measure coping but your protocol’s too long: consider the brief COPE. Int J Behav Med.

[55] Buysse DJ, Reynolds Iii CF, Monk TH, Berman SR, Kupfer DJ (1989). The Pittsburgh sleep quality index: a new instrument for psychiatric practice and research. Psychiatry Res.

[56] Watson D, Clark LA, Tellegen A (1988). Development and validation of brief measures of positive and negative affect: the PANAS scales. J Pers Soc Psychol.

[57] Bonomi AE, Patrick DL, Bushnell DM, Martin M (2000). Validation of the United States’ version of the World Health Organization Quality of Life (WHOQOL) instrument. J Clin Epidemiol.

[58] Skevington SM, Lotfy M, O’Connell KA (2004). The World Health Organization’s WHOQOL-BREF quality of life assessment: psychometric properties and results of the international field trial. A report from the WHOQOL group. Qual Life Res.

[59] Fritz CO, Morris PE, Richler JJ (2012). Effect size estimates: current use, calculations, and interpretation. J Exp Psychol Gen.

[60] Ritchie J, Spencer L, Bryman A, Burgess B (1994). Qualitative data analysis for applied policy research. Analyzing qualitative data.

[61] de Vibe M, Solhaug I, Rosenvinge JH, Tyssen R, Hanley A, Garland E. Six-year positive effects of a mindfulness-based intervention on mindfulness, coping and well-being in medical and psychology students; results from a randomized controlled trial. PLoS ONE. 2018;13(4). 10.1371/journal.pone.0196053 PMC5916495 29689081

[62] Jones DR, Lehman BJ, Noriega A, Dinnel DL. The effects of a short-term mindfulness meditation intervention on coping flexibility. Anxiety, Stress & Coping: An International Journal. 2019. 10.1080/10615806.2019.1596672 PMC6900869 30929458

[63] De Souza J, Jones LA, Rickards H (2010). Validation of self-report depression rating scales in Huntington’s disease. Mov Disord.

[64] Cosco TD, Doyle F, Ward M, McGee H (2012). Latent structure of the Hospital Anxiety and Depression Scale: a 10-year systematic review. J Psychosom Res.

[65] Dale M, Maltby J, Martucci R, Shimozaki S (2015). Factor analysis of the hospital anxiety and depression scale among a Huntington’s disease population. Mov Disord.

[66] Hocaoglu M, Gaffan E, Ho A (2012). The Huntington’s disease health-related quality of life questionnaire (HDQoL): a disease-specific measure of health-related quality of life. Clin Genet.

[67] Bogosian A, Chadwick P, Windgassen S, Norton S, McCrone P, Mosweu I (2015). Distress improves after mindfulness training for progressive MS: a pilot randomised trial. Mult Scler J.

[68] Cavalera C, Rovaris M, Mendozzi L, Pugnetti L, Garegnani M, Castelnuovo G (2019). Online meditation training for people with multiple sclerosis: a randomized controlled trial. Mult Scler J.

[69] Shechtman Z, Kiezel A (2016). Why do people prefer individual therapy over group therapy?. Int J Group Psychother.

[70] Schroevers M, Tovote K, Snippe E, Fleer J (2016). Group and individual mindfulness-based cognitive therapy (MBCT) are both effective: a pilot randomized controlled trial in depressed people with a somatic disease. Mindfulness..

[71] Stiles WB, Elliott R, Llewelyn SP, Firth-Cozens JA, Margison FR, Shapiro DA (1990). Assimilation of problematic experiences by clients in psychotherapy. Psychother Theory Res Pract Train.

[72] Sim J (2019). Should treatment effects be estimated in pilot and feasibility studies?. Pilot Feasibil Stud.

